# Potential role of gastrointestinal microbiota composition in prostate cancer risk

**DOI:** 10.1186/1750-9378-8-42

**Published:** 2013-11-04

**Authors:** E Susan Amirian, Joseph F Petrosino, Nadim J Ajami, Yanhong Liu, Martha P Mims, Michael E Scheurer

**Affiliations:** 1 Dan L Duncan Cancer Center, Baylor College of Medicine, One Baylor Plaza MS:BCM305, 77030 Houston, TX, USA; 2 Department of Pediatrics, Baylor College of Medicine, One Baylor Plaza MS:BCM305, 77030 Houston, TX, USA; 3 Department of Molcular Virology & Microbiology, Baylor College of Medicine, One Baylor Plaza MS:BCM305, 77030 Houston, TX, USA; 4 Alkek Center for Metagenomics and Microbiome Research, Baylor College of Medicine, One Baylor Plaza MS:BCM305, 77030 Houston, TX, USA; 5 Department of Medicine, Baylor College of Medicine, One Baylor Plaza MS:BCM305, 77030 Houston, TX, USA

**Keywords:** Human microbiome, Metagenome, Prostate cancer, Metabolic process

## Abstract

**Background:**

Among men in the U.S., prostate cancer is the most common cancer and the second leading cause of cancer death. Despite its prevalence, there are few established risk factors for prostate cancer. Some studies have found that intake of certain foods/nutrients may be associated with prostate cancer risk, but few have accounted for how intake and metabolic factors may interact to influence bioavailable nutrient levels and subsequent disease risk.

**Presentation of the hypothesis:**

The composition of the gastrointestinal (GI) microbiome may influence metabolism of dietary compounds and nutrients (e.g., plant phenols, calcium, choline) that may be relevant to prostate cancer risk. We, therefore, propose the hypothesis that GI microbiota may have a markedly different composition among individuals with higher prostate cancer risk. These individuals could have microbial profiles that are conducive to intestinal inflammation and/or are less favorable for the metabolism and uptake of chemopreventive agents.

**Testing the hypothesis:**

Because very little preliminary data exist on this potential association, a case–control study may provide valuable information on this topic. Such a study could evaluate whether the GI microbial profile is markedly different between three groups of individuals: healthy men, those with latent prostate cancer, and those with invasive prostate cancer. Any findings could then be validated in a larger study, designed to collect a series of specimens over time.

**Implications of the hypothesis:**

Given the plethora of information emerging from the Human Microbiome Project, this is an opportune time to explore associations between the microbiome and complex human diseases. Identification of profiles that alter the host’s risk for disease may clarify inconsistencies in the literature on dietary factors and cancer risk, and could provide valuable targets for novel cancer prevention strategies.

## Background

Prostate cancer is the most common cancer among men in the U.S. [1]. In 2012, approximately 241,740 new diagnoses and 28,170 prostate cancer-related deaths were expected in the U.S. alone (global incidence of 27.9 cases per 100,000) [1,2]. Lifetime risk for prostate cancer is estimated to be 16%, and the median age at diagnosis is 67 years [1].

Despite its prevalence, there are few established risk factors for prostate cancer [3]. According to twin studies, a proportion of cases (10-40%) may be explained by genetic factors [3-5]. However, dietary and lifestyle factors may also influence prostate cancer susceptibility [3,6]. Intake of red meat [7-10], dairy products [11,12], eggs [9,13,14], green tea [15,16], calcium [17-20], lycopene [21-23], selenium [6,24], and fish oil [4,25] have all been examined in relation to prostate cancer risk with relatively inconsistent results. The inconsistency of these findings may partly be due to the use of food intake measures as surrogates for bioavailable micronutrient levels, resulting in some misclassification of nutrient/metabolite exposures [26,27]. Differing levels of nutrient metabolism and absorption from foods between study participants could bias the results of intake studies. Bioavailable micronutrient levels are often determined by complex interactions between intake and metabolism, which are generally not accounted for in such studies.

While many studies have examined self-reported dietary intake of certain foods or biomarkers of specific nutrient levels, few studies have focused on the interactions between how intake and metabolic factors may be working together to influence bioavailable nutrient levels and, potentially, disease risk. The studies that have examined interactions have mostly investigated how genetic variation may affect metabolism or intake (i.e., [28-30]). For example, one recent study investigated the effect of a single-nucleotide polymorphism (rs4988235) in the lactase (*LCT*) gene on dairy intake, blood analytes, and prostate cancer risk [29]. Although they did not find a significant association with prostate cancer susceptibility, they did report that this variant was correlated with milk intake, with the genotype that confers lower tolerance of lactose-containing foods being associated with lower dairy intake. Another study found that obesity (and its inflammatory sequelae) may modify the impact of arachidonic acid metabolism gene polymorphisms on prostate cancer risk [30]. Nevertheless, genetic factors are only one component of what determines the ability to absorb, convert, and retain dietary nutrients [31-33].

Bioavailable micronutrient levels are not only dependent upon metabolism-related genetic profiles, but are also partly determined by the composition of one’s gastrointestinal (GI) microbiota and the metabolic profiles of these GI microorganisms [31,32,34-37]. In fact, the relationship between the GI microbiome and dietary factors is bidirectional-- diet influences the composition of the GI microbiome and the GI microbiome affects the digestion and metabolism of dietary factors [37]. Interactions between intake and many species of microbes in the host GI tract have already been well documented [31,32,38,39], and there are several excellent comprehensive review articles currently available on this topic [36,37,39]. Here, we will highlight a few examples of such interactions to show that the GI microbiome and its related metabolic properties can potentially be highly relevant to prostate cancer risk.

Microbial metabolism in the gut can affect dairy product digestion [40], influence the composition of bioactive fatty acids in host adipose tissue [35,41], alter dietary phytochemical digestion/uptake [33], and contribute to the generation of carcinogenic metabolites and inflammation [33,42-45], among many other effects. For example, *Lactobacillus acidophilus*, is commonly used for probiotic supplementation, as it may aid in lactose digestion [46,47], and *Lactobacillus salivarius* can help kill *Listeria*, possibly preventing food-borne illness. Another example involves phenolic compounds from tea, coffee, and other plant-based dietary sources [33]. Some species of GI microbes help digest phenols into biologically active metabolites, which are more readily absorbed by the host [33,48,49]. Resveratrol is one such phenol that has been shown to have anti-inflammatory effects by altering eicosanoid production and inhibiting cytokines such as PTGS2, IL6, and TNF [33,50]. By providing additional enzymatic action, these and other GI microorganism can have major impacts on the host’s digestive process. Some enzymes, such as β-glucuronidases, that may be of particular relevance to prostate cancer development are involved in conjugation and de-conjugation of sex hormones, such as estrogen [51-53]. In fact, fecal microbial richness and alpha diversity have previously been associated with total urinary estrogen levels [52]. In addition to these examples, a few other illustrations of potentially relevant relationships between xenobiotics and GI microorganisms are provided in Table 1.

**Table 1:** Examples of known associations between components of the gastrointestinal microbiome and xenobiotic compounds*

**Xenobiotics**	**Xenobiotic Function**	**Metabolizing Bacteria**	**Effect**	**Reference**
Selenium	Antioxidant	Unknown	Partial sequestration, limiting availability to host	[54]
2-Amino-3-methylimidazo [4,5-f]quinoline (IQ)	Carcinogenic heterocyclic amine, food-borne	*Bacteroides, Clostridium, Escherichia*	Degraded into 7-hydroxy-IQ (direct mutagen) by β-glucuronidase	[55][56]
Diadzein	Soy phytoestrogen	Unknown	Metabolized into equol or non-estrogenic metabolites	[57]
Methylmercuric chloride	Mercuric toxicity	Unknown	Reduction of mercuric tissue content	[58]

*Examples identified through PharmacoMicrobiomics: The Drug-Microbiome Portal (http://pharmacomicrobiomics.com/).

A recent review article provided further rationale behind why the GI bacterial community should be viewed as a biodynamic system that interacts with its living environment [38] and may, thus, affect disease risk. This recent article focused on the hypothesis that GI microbes could influence prostate cancer risk based on the presence of isoflavone-metabolizing, equol-producing bacteria. Equol, which has anti-androgenic properties, is being tested as a potential chemopreventive agent. The idea of *Slackia* sp. NATTS strain bacteria metabolizing daidzein into equol, which may, subsequently, influence prostate cancer susceptibility, provides yet another example of how the GI microbiome could impact digestion and potentially have downstream systemic effects. With a ratio of about 10 microbes for each eukaryotic cell in the human body [59,60], it is likely that the human microbiome has major physiological and metabolic impacts that we have yet to uncover.

Plotter and Blaser have suggested that human cancers should be considered in the milieu of host-microbiome interactions. They previously described three paradigms relating how the microbiome may be involved in cancer development and pathogenesis. The first involves constituents of the microbiome having inflammatory effects in a lumenal organ. The second paradigm revolves around the metabolic effects of the host’s GI microbiome indirectly contributing to distal malignancies via the human estrobolome. The estrobolome is defined as the set of enteric bacterial genes which code for proteins involved in estrogen metabolism. This paradigm may be particularly relevant to our proposed hypothesis due to the reported associations between estrogens and prostate cancer risk [53,61]. The third paradigm is related to the alteration of clinical latency preceding malignancies.

Additionally, there are several specific mechanisms (some of which could fall under one or more of the aforementioned paradigms) through which the GI microbiome could have downstream effects on cancer risk, including competitive inhibition of pathogenic bacteria, production of antibacterial compounds (i.e., bacteriocins) and acids, gene transference between food-borne microbes and members of the GI microbiota, and modulation of the host’s immune system [37,62]. The exact mechanisms by which the GI microbiome may distally affect cancer risk is likely to be different depending on the cancer site. Nevertheless, the first step to determining how influential the GI microbiome may be in prostate cancer development is to assess whether there are key distinctions in the microbial profiles of men who do and do not develop aggressive disease.

## Presentation of the hypothesis

Our hypothesis is that gastrointestinal microbiota may have a markedly different composition among individuals with higher prostate cancer risk. It is expected that men who are more susceptible to the development of aggressive disease will have similarities in their microbial/metabolic profiles that diverge from the profiles of healthy men. With regard to the microbial profiles of interest, our hypothesis is not, *per se,* contingent upon the taxonomic composition of the GI microbiome of low- versus high-risk individuals, but rather the different metabolic and functional profiles represented by the GI microbial community. Given that different taxa of bacteria can have similar metabolic effects, both taxonomic and metabolic profiles should be identified and compared between high- and low-risk men. Furthermore, because of the varied effects that intestinal bacteria can have on the host, many of which are still being uncovered, functional studies and research into the specific mechanisms of action will be necessary to explain any microbial or metabolic differences found. Individuals with higher prostate cancer susceptibility may potentially have microbial profiles that are more conducive to intestinal inflammation and/or less favorable for the metabolism and uptake of chemopreventive agents, certain micronutrients, etc.

## Testing the hypothesis

Testing our hypothesis may involve a distinct set of challenges. Although a case–control design seems appropriate, a prospective study would better distinguish between cancer-induced changes in the microbiome, as opposed to changes that may play an etiologic or augmentative role in carcinogenesis (Table 2). Khan et al. posit that there are at least five mechanisms through which the microbiome could be altered by cancer development [63]. Changes to cell surface receptors and ligands may prevent a microbe from selectively binding to certain host cells. The process of carcinogenesis may also involve immunological alterations, which can prevent recognition of pathogenic versus symbiotic bacteria. Further hormonal, anatomical, and enzymatic changes that occur in the host’s body during cancer development may produce a host environment that inhibits survival of one microbe over another. Additional concerns involve changes that may occur in the GI microbial profile due to post-diagnostic alterations in diet among cases during therapy.

**Table 2:** Strengths and limitations of epidemiologic study designs for examining the associations between the gastrointestinal microbiome and prostate cancer risk

**Study design**	**Strengths**	**Limitations**
*Case–control:*	● Quick	● Cannot truly establish temporality or differentiate between cancer-induced and pre-cancerous changes in the GI microbiome
Diagnostically-confirmed latent and invasive prostate cancer cases compared to each other and to matched controls	● Relatively inexpensive	
	● No follow-up required	
		● Difficult to obtain appropriate control group
*Prospective Cohort:*	● Ability to assess changes from multiple samples as individuals transition from healthy to cancerous state	● Need very large sample size to be able to obtain enough incident prostate cancer cases
Large cohort of older men followed over time with regular assessments of GI microbiome and prostate cancer status		
		● Need long follow up time
	● Can obtain data on incident cases	● Extremely expensive
		● Potential biases due to loss to follow
	● Can continue to obtain data throughout course of treatment and progression to assess post-diagnostic longitudinal and treatment-related changes	
	● Can evaluate mortality as an end point	
*Retrospective Cohort:*	● Likely to have clearer temporality between assessment of microbial/metabolic profile and prostate cancer development than in a case–control study	● Requires availability of previously collected and appropriately preserved samples (or data)
Previously collected samples on a large cohort of men, who were healthy at baseline, assessed for current prostate cancer status		
		● Participants must have been cancer-free at time of sample collection
	● Less expensive than prospective cohort study	● Multiple longitudinal samples are unlikely to be available

Another concern regarding the temporality of potential associations between the microbiome and cancer development relates to the “driver-passenger model” that has been proposed for colorectal cancer [64]. Tjalsma et al. posit that colorectal carcinogenesis may be spurred by “driver” bacteria, which can initially induce DNA damage, and are later replaced by “passenger” bacteria that could either delay or enhance tumorogeneis. They suggest that the changing microenvironment surrounding the growth of the tumor may alter selective pressures and, thus, result in the driver bacteria being outcompeted by passenger bacteria (which are defined as commensal organisms that may have tumor promoting or suppressing properties). This driver-passenger model of the involvement of the GI microbiome in colorectal cancer development is probably less applicable to cancers in tissues that have little direct exposure to the microbiome, such as the prostate. Nonetheless, it is possible that physiological changes that occur after the development of prostate cancer may, in turn, have an indirect impact on the composition of the GI microbiome. As a result, it is important that the issue of temporality be considered in any study of the possible associations between the GI microbiome and prostate cancer risk.

While knowledge of microbial changes that occur *due to* cancer development may be useful as potential diagnostic or screening tools, identification of changes in gastrointestinal microbiota that increase one’s *risk* for invasive cancer would provide a key opportunity for cancer prevention. A prospective study design, through expensive and time-consuming, may afford opportunities to study both these topics, if a large enough cohort of men could be recruited and followed. Exploring the composition of the GI microbiome in relation to prostate cancer risk over time may clarify the findings of previous studies that have inconsistently reported associations between intake of various foods/nutrients and prostate cancer susceptibility by better encompassing the complex set of interactions involved in digestion/metabolism. However, a longitudinal study may present several obstacles related to feasibility, given the incidence of prostate cancer among the general population, the cost of repeated evaluations of microbial profiles, and the need to successfully follow the participants over time while minimizing loss to follow up.

Sample collection could pose another challenge for a longitudinal study on this topic. Many protocols require that stool samples be kept on ice and returned to the lab within 24 hours of collection. A prospective study that requires participants to collect stool, pack it, and return it to the lab within one day may have high loss to follow up, which could be differential between those who go on to develop prostate cancer versus those who do not. This type of selection bias would impact the study findings. Thus, procedures should be streamlined, detailed instructions must be given to participants, and appropriate study incentives should be provided. Ideally, sample collection and processing should follow the protocols set forth and established by The Human Microbiome Project [65,66].

Given the feasibility-related issues that may be associated with a longitudinal study on the GI microbiome and prostate cancer susceptibility, initial studies may realistically need to be retrospective to determine whether this topic is a fruitful area of research. Because little preliminary data exist on this potential association, a case–control study (recruiting incident cases) may provide valuable information, despite its limitations. The GI microbial profiles of healthy men can be compared to those with latent prostate cancer and those with invasive prostate cancer. Alternatively, in a prospective cohort including only diagnostically-confirmed cases, the GI microbiome can feasibly be examined in relation to prostate cancer survival over time among men with aggressive disease to assess its prognostic value.

## Implications of the hypothesis

Investigating the role of the human microbiome in the etiology of complex multifactorial conditions, like cancer, is still a relatively new field. Much research to date has focused on profiling the composition of the microbiome across the human body, rather than exploring associations with disease. The recent literature contains several profiles of the bacterial diversity across the human digestive tract [67-70]. However, *inter*-individual differences in GI microbiome composition are a source of genetic/metabolic variation that warrant thorough analysis as potential predictors of disease susceptibility.

Even if the hypothesis proposed here is consistently supported by our and other similar studies, establishing causation for this association will likely be relatively difficult. Any differences in the GI microbiome detected between low- and high-risk (or healthy and diseased) men are likely to be neither sufficient nor necessary for the development of malignancy. Furthermore, the association of interest is essentially a network of interactions between the GI microbiome, dietary factors, and the host’s other environmental and genetic susceptibility factors. Studying such interactive relationships between interconnected exogenous and endogenous factors has always been challenging, but as the revolutionary new field of *molecular pathologic epidemiology* continues to evolve, the pathogenic processes behind complex diseases can slowly be uncovered [71,72].

Molecular pathologic epidemiology can be described as the study of the interactive relationships between lifestyle/dietary factors and molecular tumoral characteristics on the development or progression of a specific molecular subtype of cancer [72]. Because this field will attempt to examine more homogeneous and specific outcomes, while also accounting for the network of biological interactions inevitably at play in the disease process, etiologic relationships that have long eluded us because of the heterogeneity of the case definition (or because of the reductionist approach of simply examining main effects) may begin to be revealed. It follows that simple standards, such as Koch’s postulates or Hill’s criteria, which have previously been used for establishing causality, are unlikely to be entirely applicable to studies such as the one proposed here (which will be focused on the multiple networks of interactions constituted by the potential relationships between the GI microbiome and prostate cancer) [73]. Therefore, as the field of molecular pathologic epidemiology grows, the criteria by which we assess causality must also evolve to incorporate a more systemic approach.

Nevertheless, given the available information from the Human Microbiome Project [74,75], this is an opportune time to clarify associations between the microbiome and complex diseases [66]. Identification of profiles that alter the host’s disease risk may clarify inconsistencies in the literature on dietary factors and cancer risk, and will likely provide novel targets for cancer prevention strategies and personalized medicine [33]. Such strategies may involve personalized probiotic and vitamin/mineral supplementation, fecal transplant [76-78], or the use of antibiotics to achieve a more favorable microbial profile among men whose GI microbiota may support a predisposition to invasive prostate cancer.

## Competing interests

The authors declare no competing interests.

## Authors’ contributions

All authors were involved in drafting and critically revising this article. All authors read and approved the final manuscript.

## References

[B1] SEER stat fact sheets: prostate[http://www.seer.cancer.gov/statfacts/html/prost.html]

[B2] Brenner AV Linet MS Fine HA Shapiro WR Selker RG Black PM Inskip PD History of allergies and autoimmune diseases and risk of brain tumors in adults Int J Cancer 2002 99 252 259 10.1002/ijc.10320 11979441

[B3] Wilson KM Giovannucci EL Mucci LA Lifestyle and dietary factors in the prevention of lethal prostate cancer Asian J Androl 2012 14 365 374 10.1038/aja.2011.142 22504869 PMC3720164

[B4] Astorg P Dietary N-6 and N-3 polyunsaturated fatty acids and prostate cancer risk: a review of epidemiological and experimental evidence Cancer Causes Control 2004 15 367 386 15141138 10.1023/B:CACO.0000027498.94238.a3

[B5] Page WF Braun MM Partin AW Caporaso N Walsh P Heredity and prostate cancer: a study of World War II veteran twins Prostate 1997 33 240 245 10.1002/(SICI)1097-0045(19971201)33:4<240::AID-PROS3>3.0.CO;2-L 9397195

[B6] Sonn GA Aronson W Litwin MS Impact of diet on prostate cancer: a review Prostate Cancer Prostatic Dis 2005 8 304 310 10.1038/sj.pcan.4500825 16130015

[B7] Punnen S Hardin J Cheng I Klein EA Witte JS Impact of meat consumption, preparation, and mutagens on aggressive prostate cancer PLoS One 2011 6 e27711 10.1371/journal.pone.0027711 22132129 PMC3223211

[B8] Major JM Cross AJ Watters JL Hollenbeck AR Graubard BI Sinha R Patterns of meat intake and risk of prostate cancer among African-Americans in a large prospective study Cancer Causes Control 2011 22 1691 1698 10.1007/s10552-011-9845-1 21971816 PMC3403708

[B9] Richman EL Kenfield SA Stampfer MJ Giovannucci EL Chan JM Egg, red meat, and poultry intake and risk of lethal prostate cancer in the prostate-specific antigen-era: incidence and survival Cancer Prev Res (Phila) 2011 4 2110 2121 10.1158/1940-6207.CAPR-11-0354 21930800 PMC3232297

[B10] Alexander DD Mink PJ Cushing CA Sceurman B A review and meta-analysis of prospective studies of red and processed meat intake and prostate cancer Nutr J 2010 9 50 10.1186/1475-2891-9-50 21044319 PMC2987772

[B11] Newmark HL Heaney RP Dairy products and prostate cancer risk Nutr Cancer 2010 62 297 299 10.1080/01635580903407221 20358466

[B12] Lampe JW Dairy products and cancer J Am Coll Nutr 2011 30 464S 470S 10.1080/07315724.2011.10719991 22081693

[B13] Richman EL Kenfield SA Stampfer MJ Giovannucci EL Zeisel SH Willett WC Chan JM Choline intake and risk of lethal prostate cancer: incidence and survival Am J Clin Nutr 2012 96 855 863 10.3945/ajcn.112.039784 22952174 PMC3441112

[B14] Xie B He H No association between egg intake and prostate cancer risk: a meta-analysis Asian Pac J Cancer Prev 2012 13 4677 4681 10.7314/APJCP.2012.13.9.4677 23167401

[B15] Montague JA Butler LM Wu AH Genkinger JM Koh WP Wong AS Wang R Yuan JM Yu MC Green and black tea intake in relation to prostate cancer risk among Singapore Chinese Cancer Causes Control 2012 23 1635 1641 10.1007/s10552-012-0041-8 22864870 PMC3695613

[B16] Ozten-Kandas N Bosland MC Chemoprevention of prostate cancer: natural compounds, antiandrogens, and antioxidants - in vivo evidence J Carcinog 2011 10 27 10.4103/1477-3163.90438 22190869 PMC3243088

[B17] Chan JM Giovannucci EL Dairy products, calcium, and vitamin D and risk of prostate cancer Epidemiol Rev 2001 23 87 92 10.1093/oxfordjournals.epirev.a000800 11588859

[B18] Gao X LaValley MP Tucker KL Prospective studies of dairy product and calcium intakes and prostate cancer risk: a meta-analysis J Natl Cancer Inst 2005 97 1768 1777 10.1093/jnci/dji402 16333032

[B19] Schwartz GG Skinner HG A prospective study of total and ionized serum calcium and time to fatal prostate cancer Cancer Epidemiol Biomarkers Prev 2012 21 1768 1773 10.1158/1055-9965.EPI-12-0585 22914529

[B20] Chan JM Stampfer MJ Ma J Gann PH Gaziano JM Giovannucci EL Dairy products, calcium, and prostate cancer risk in the Physicians’ health study Am J Clin Nutr 2001 74 549 554 11566656 10.1093/ajcn/74.4.549

[B21] Key TJ Appleby PN Allen NE Travis RC Roddam AW Jenab M Egevad L Tjonneland A Johnsen NF Overvad K Plasma carotenoids, retinol, and tocopherols and the risk of prostate cancer in the European prospective investigation into cancer and nutrition study Am J Clin Nutr 2007 86 672 681 17823432 10.1093/ajcn/86.3.672

[B22] Wu K Erdman JW Jr Schwartz SJ Platz EA Leitzmann M Clinton SK DeGroff V Willett WC Giovannucci E Plasma and dietary carotenoids, and the risk of prostate cancer: a nested case–control study Cancer Epidemiol Biomarkers Prev 2004 13 260 269 10.1158/1055-9965.EPI-03-0012 14973107

[B23] Teodoro AJ Oliveira FL Martins NB Maia GA Martucci RB Borojevic R Effect of lycopene on cell viability and cell cycle progression in human cancer cell lines Cancer Cell Int 2012 12 36 10.1186/1475-2867-12-36 22866768 PMC3492052

[B24] Hurst R Hooper L Norat T Lau R Aune D Greenwood DC Vieira R Collings R Harvey LJ Sterne JA Selenium and prostate cancer: systematic review and meta-analysis Am J Clin Nutr 2012 96 111 122 10.3945/ajcn.111.033373 22648711

[B25] Sala-Vila A Calder PC Update on the relationship of fish intake with prostate, breast, and colorectal cancers Crit Rev Food Sci Nutr 2011 51 855 871 10.1080/10408398.2010.483527 21888535

[B26] Dennis LK Snetselaar LG Smith BJ Stewart RE Robbins ME Problems with the assessment of dietary fat in prostate cancer studies Am J Epidemiol 2004 160 436 444 10.1093/aje/kwh243 15321840

[B27] Wei MY Giovannucci EL Lycopene, tomato products, and prostate cancer incidence: a review and reassessment in the PSA screening Era J Oncol 2012 2012 271063 22690215 10.1155/2012/271063 PMC3368367

[B28] Joshi AD Corral R Catsburg C Lewinger JP Koo J John EM Ingles SA Stern MC Red meat and poultry, cooking practices, genetic susceptibility and risk of prostate cancer: results from a multiethnic case–control study Carcinogenesis 2012 33 2108 2118 10.1093/carcin/bgs242 22822096 PMC3584966

[B29] Travis RC Appleby PN Siddiq A Allen NE Kaaks R Canzian F Feller S Tjonneland A Fons Johnsen N Overvad K Genetic variation in the lactase gene, dairy product intake and risk for prostate cancer in the European prospective investigation into cancer and nutrition Int J Cancer 2012 132 1901 1910 22965418 10.1002/ijc.27836 PMC3594976

[B30] Amirian ES Ittmann MM Scheurer ME Associations between arachidonic acid metabolism gene polymorphisms and prostate cancer risk Prostate 2011 71 1382 1389 10.1002/pros.21354 21308720 PMC7339922

[B31] Tilg H Obesity, metabolic syndrome, and microbiota: multiple interactions J Clin Gastroenterol 2010 44 Suppl 1 S16 S18 20535027 10.1097/MCG.0b013e3181dd8b64

[B32] Tremaroli V Backhed F Functional interactions between the gut microbiota and host metabolism Nature 2012 489 242 249 10.1038/nature11552 22972297

[B33] Macdonald RS Wagner K Influence of dietary phytochemicals and microbiota on colon cancer risk J Agric Food Chem 2012 [Epub ahead of print] PMID: 22632581 10.1021/jf204230r 22632581

[B34] Musso G Gambino R Cassader M Interactions between gut microbiota and host metabolism predisposing to obesity and diabetes Annu Rev Med 2011 62 361 380 10.1146/annurev-med-012510-175505 21226616

[B35] Musso G Gambino R Cassader M Obesity, diabetes, and gut microbiota: the hygiene hypothesis expanded? Diabetes Care 2010 33 2277 2284 10.2337/dc10-0556 20876708 PMC2945175

[B36] Haiser HJ Turnbaugh PJ Developing a metagenomic view of xenobiotic metabolism Pharmacol Res: J Italian Pharmacol Soc 2013 69 21 31 10.1016/j.phrs.2012.07.009 PMC3526672 22902524

[B37] Dutton RJ Turnbaugh PJ Taking a metagenomic view of human nutrition Curr Opin Clin Nutr 2012 15 448 454 10.1097/MCO.0b013e3283561133 22878238

[B38] Akaza H Prostate cancer chemoprevention by soy isoflavones: role of intestinal bacteria as the "second human genome" Cancer Sci 2012 103 969 975 10.1111/j.1349-7006.2012.02257.x 22372745 PMC7685082

[B39] Saad R Rizkallah MR Aziz RK Gut pharmacomicrobiomics: the tip of an iceberg of complex interactions between drugs and gut-associated microbes Gut Pathogens 2012 4 16 10.1186/1757-4749-4-16 23194438 PMC3529681

[B40] Masood MI Qadir MI Shirazi JH Khan IU Beneficial effects of lactic acid bacteria on human beings Crit Rev Microbiol 2011 37 91 98 10.3109/1040841X.2010.536522 21162695

[B41] Backhed F Ding H Wang T Hooper LV Koh GY Nagy A Semenkovich CF Gordon JI The gut microbiota as an environmental factor that regulates fat storage Proc Natl Acad Sci U S A 2004 101 15718 15723 10.1073/pnas.0407076101 15505215 PMC524219

[B42] Russell SL Finlay BB The impact of gut microbes in allergic diseases Curr Opin Gastroenterol 2012 28 563 569 10.1097/MOG.0b013e3283573017 23010680

[B43] Arthur JC Perez-Chanona E Muhlbauer M Tomkovich S Uronis JM Fan TJ Campbell BJ Abujamel T Dogan B Rogers AB Intestinal inflammation targets cancer-inducing activity of the microbiota Science 2012 338 120 123 10.1126/science.1224820 22903521 PMC3645302

[B44] Graessler J Qin Y Zhong H Zhang J Licinio J Wong ML Xu A Chavakis T Bornstein AB Ehrhart-Bornstein M Metagenomic sequencing of the human gut microbiome before and after bariatric surgery in obese patients with type 2 diabetes: correlation with inflammatory and metabolic parameters Pharmacogenomics J 2012 doi: 10.1038/tpj.2012.43. [Epub ahead of print] PMID: 23032991 10.1038/tpj.2012.43 23032991

[B45] Iebba V Nicoletti M Schippa S Gut microbiota and the immune system: an intimate partnership in health and disease Int J Immunopathol Pharmacol 2012 25 823 833 23298474 10.1177/039463201202500401

[B46] Sanders ME Klaenhammer TR Invited review: the scientific basis of lactobacillus acidophilus NCFM functionality as a probiotic J Dairy Sci 2001 84 319 331 10.3168/jds.S0022-0302(01)74481-5 11233016

[B47] Montes RG Bayless TM Saavedra JM Perman JA Effect of milks inoculated with lactobacillus acidophilus or a yogurt starter culture in lactose-maldigesting children J Dairy Sci 1995 78 1657 1664 10.3168/jds.S0022-0302(95)76790-X 8786251

[B48] Sekirov I Russell SL Antunes LC Finlay BB Gut microbiota in health and disease Physiol Rev 2010 90 859 904 10.1152/physrev.00045.2009 20664075

[B49] Russell W Duthie G Plant secondary metabolites and gut health: the case for Phenolic acids Proc Nutr Soc 2011 70 389 396 10.1017/S0029665111000152 21781364

[B50] Namasivayam N Chemoprevention in experimental animals Ann N Y Acad Sci 2011 1215 60 71 10.1111/j.1749-6632.2010.05873.x 21261642

[B51] Plottel CS Blaser MJ Microbiome and malignancy Cell host & microbe 2011 10 324 335 10.1016/j.chom.2011.10.003 22018233 PMC3264051

[B52] Flores R Shi J Fuhrman B Xu X Veenstra TD Gail MH Gajer P Ravel J Goedert JJ Fecal microbial determinants of fecal and systemic estrogens and estrogen metabolites: a cross-sectional study J Transl Med 2012 10 253 10.1186/1479-5876-10-253 23259758 PMC3552825

[B53] Cavalieri E Rogan E The molecular etiology and prevention of estrogen-initiated cancers Mol Aspects Med 2013 doi: 10.1016/j.mam.2013.08.002. [Epub ahead of print] PMID: 23994691 10.1016/j.mam.2013.08.002 PMC3938998 23994691

[B54] Kasaikina MV Kravtsova MA Lee BC Seravalli J Peterson DA Walter J Legge R Benson AK Hatfield DL Gladyshev VN Dietary selenium affects host selenoproteome expression by influencing the gut microbiota FASEB J: Publ Fed Am Soc Exp Biol 2011 25 2492 2499 10.1096/fj.11-181990 PMC3114522 21493887

[B55] Humblot C Combourieu B Vaisanen ML Furet JP Delort AM Rabot S 1H Nuclear magnetic resonance spectroscopy-based studies of the metabolism of food-borne carcinogen 2-amino-3-methylimidazo[4,5-f]quinoline by human intestinal microbiota Appl Environ Microb 2005 71 5116 5123 10.1128/AEM.71.9.5116-5123.2005 PMC1214681 16151094

[B56] Humblot C Murkovic M Rigottier-Gois L Bensaada M Bouclet A Andrieux C Anba J Rabot S Beta-glucuronidase in human intestinal microbiota is necessary for the colonic genotoxicity of the food-borne carcinogen 2-amino-3-methylimidazo[4,5-f]quinoline in rats Carcinogenesis 2007 28 2419 2425 10.1093/carcin/bgm170 17660508

[B57] Rafii F Jackson LD Ross I Heinze TM Lewis SM Aidoo A Lyn-Cook L Manjanatha M Metabolism of daidzein by fecal bacteria in rats Comparat Med 2007 57 282 286 17605343

[B58] Rowland IR Davies MJ Evans JG Tissue content of mercury in rats given methylmercuric chloride orally: influence of intestinal flora Arch Environ health 1980 35 155 160 10.1080/00039896.1980.10667485 7387196

[B59] Li K Bihan M Yooseph S Methe BA Analyses of the microbial diversity across the human microbiome PLoS One 2012 7 e32118 10.1371/journal.pone.0032118 22719823 PMC3374608

[B60] Goodman AL Gordon JI Our unindicted coconspirators: human metabolism from a microbial perspective Cell Metab 2010 12 111 116 10.1016/j.cmet.2010.07.001 20674856 PMC2935662

[B61] Carruba G Estrogen and prostate cancer: an eclipsed truth in an androgen-dominated scenario J Cell Biochem 2007 102 899 911 10.1002/jcb.21529 17786930

[B62] Commane D Hughes R Shortt C Rowland I The potential mechanisms involved in the anti-carcinogenic action of probiotics Mutat Res 2005 591 276 289 10.1016/j.mrfmmm.2005.02.027 16095630

[B63] Khan AA Shrivastava A Khurshid M Normal to cancer microbiome transformation and its implication in cancer diagnosis Biochim Biophys Acta 1826 2012 331 337 10.1016/j.bbcan.2012.05.005 22683403

[B64] Tjalsma H Boleij A Marchesi JR Dutilh BE A bacterial driver-passenger model for colorectal cancer: beyond the usual suspects Nat Rev Microbiol 2012 10 575 582 10.1038/nrmicro2819 22728587

[B65] NIH human microbiome project sampling, sequencing, and analysis protocols[http://www.hmpdacc.org/tools_protocols/tools_protocols.php]

[B66] Dave M Higgins PD Middha S Rioux KP The human gut microbiome: current knowledge, challenges, and future directions Transl Res: J Lab Clin Med 2012 160 246 257 10.1016/j.trsl.2012.05.003 22683238

[B67] Segata N Haake SK Mannon P Lemon KP Waldron L Gevers D Huttenhower C Izard J Composition of the adult digestive tract bacterial microbiome based on seven mouth surfaces, tonsils, throat and stool samples Genome Biol 2012 13 R42 10.1186/gb-2012-13-6-r42 22698087 PMC3446314

[B68] Stearns JC Lynch MD Senadheera DB Tenenbaum HC Goldberg MB Cvitkovitch DG Croitoru K Moreno-Hagelsieb G Neufeld JD Bacterial biogeography of the human digestive tract Sci Rep 2011 1 170 22355685 10.1038/srep00170 PMC3240969

[B69] Huse SM Ye Y Zhou Y Fodor AA A core human microbiome as viewed through 16S rRNA sequence clusters PLoS One 2012 7 e34242 10.1371/journal.pone.0034242 22719824 PMC3374614

[B70] Antonopoulos DA Huse SM Morrison HG Schmidt TM Sogin ML Young VB Reproducible community dynamics of the gastrointestinal microbiota following antibiotic perturbation Infect Immun 2009 77 2367 2375 10.1128/IAI.01520-08 19307217 PMC2687343

[B71] Ogino S Stampfer M Lifestyle factors and microsatellite instability in colorectal cancer: the evolving field of molecular pathological epidemiology J Natl Cancer Inst 2010 102 365 367 10.1093/jnci/djq031 20208016 PMC2841039

[B72] Ogino S Chan AT Fuchs CS Giovannucci E Molecular pathological epidemiology of colorectal neoplasia: an emerging transdisciplinary and interdisciplinary field Gut 2011 60 397 411 10.1136/gut.2010.217182 21036793 PMC3040598

[B73] Plowright RK Sokolow SH Gorman ME Daszak P Foley JE Causal inference in disease ecology: investigating ecological drivers of disease emergence Front Ecol Environ 2008 6 420 429 10.1890/070086

[B74] Morgan XC Segata N Huttenhower C Biodiversity and functional genomics in the human microbiome Trends Genet 2012 29 51 58 23140990 10.1016/j.tig.2012.09.005 PMC3534939

[B75] Conlan S Kong HH Segre JA Species-level analysis of DNA sequence data from the NIH human microbiome project PLoS One 2012 7 e47075 10.1371/journal.pone.0047075 23071716 PMC3468466

[B76] Vindigni SM Broussard EK Surawicz CM Alteration of the intestinal microbiome: fecal microbiota transplant and probiotics for clostridium difficile and beyond Expert Rev Gastroenterol Hepatol 2013 7 615 628 10.1586/17474124.2013.832501 24070153

[B77] Aroniadis OC Brandt LJ Fecal microbiota transplantation: past, present and future Curr Opin Gastroenterol 2013 29 79 84 10.1097/MOG.0b013e32835a4b3e 23041678

[B78] Paasche S Fecal microbiota transplantation: an innovative approach to treating clostridium difficile disease JAAPA: J Am Acad Physic Assistants 2013 26 46 49 10.1097/01.jaa.0000432570.98817.16 24049940

